# Conventional and Organic Wheat Germ Have Distinct Physiological Effects in the Tobacco Hornworm, *Manduca Sexta*: Use of *Black* Mutant Assay to Detect Environmental Juvenoid Activity of Insect Growth Regulators

**DOI:** 10.3389/finsc.2021.744847

**Published:** 2021-10-21

**Authors:** Jivonsha Ffrench, Jaime Tracewell, Yuichiro Suzuki

**Affiliations:** Department of Biological Sciences, Wellesley College, Wellesley, MA, United States

**Keywords:** insect growth regulator (IGR), juvenile hormone (JH), bioassay, *Manduca sexta* (Insecta), growth

## Abstract

Stored grains used in artificial diets are often treated with insecticides to control infestation by pests. In recent years, insect growth regulators (IGRs) have become an increasingly popular form of insect pest control in agricultural settings. Most IGRs specifically target insects by either disrupting their endocrine system or their chitin synthesis. One type of IGRs comprises of chemical analogs of juvenile hormone (JH), a major hormone involved in growth and development of insects. Here we demonstrate that conventional wheat germ contains JH activity and impacts growth and development of the tobacco hornworm, *Manduca sexta*. Feeding diet containing conventional wheat germ delayed the timing of metamorphosis in wildtype larvae by extending the duration of the final instar. Diet with conventional wheat germ also inhibited melanization of the *black* mutant larvae and induced the expression of the JH response gene, *Krüppel homolog 1*. We demonstrate that the *black* mutant bioassay is a sensitive assay that can determine the amount of JH activity in stored grains and suggest that this assay may offer a quick and reliable assay to determine the amount of environmental juvenoids. Researchers are urged to use caution when purchasing stored grains for mass-rearing of research insects.

## Introduction

Stored grains are often treated with insecticides to control infestation by pests. Insect growth regulators (IGRs) have become a popular replacement for organophosphorus, pyrethroid and carbamate insecticides that often impact human health (1, 2). The major benefit of using IGRs stems from their specificity to insects (2). IGRs mainly target insects by interfering with their development, growth and chitin synthesis (2, 3). Many IGRs act as chemical analogs of two major insect hormones, the sesquiterpenoid juvenile hormone (JH) and the molting hormone, ecdysteroids (3). Application of such hormonal IGRs can disrupt major developmental events, such as metamorphosis, molting and reproduction (3).

JH plays several major roles in insect development and growth. During the early larval instars, the presence of JH prevents larvae from molting into a pupa (4). Thus, high levels of JH ensures that a larva molts into another larva. In the final larval instar, JH titers drop dramatically, signaling the larvae to undergo metamorphosis. JH is also involved in regulating the growth of many insects. In the final instar of the tobacco hornworm, *Manduca sexta*, prothoracicotropic hormone (PTTH) stimulates the prothoracic glands (5), which in turn release ecdysteroids that cause the larva to cease feeding, clear its gut and initiate the wandering behavior in search of a pupation site (6). JH must be cleared for the brain to become competent to secrete PTTH (7). Topical application of JH in the final instar therefore prevents metamorphosis and allows larvae to continue feeding and grow to a larger size (8, 9).

Despite its importance, detection of JH is notoriously challenging due to its low titers and chemical structure. Although several different assays have been developed [e.g., (10, 11)], here we focus on two sensitive and relatively easy methods to assay JH levels: the determination of the expression of a JH response gene and the *black* mutant bioassay. JH binds to the JH receptor, a basic helix-loop-helix-Per-Arnt-Sim domain protein receptor called Methoprene-tolerant (12, 13). Once bound by JH, Methoprene-tolerant binds to the promoter of the JH-response gene encoding a transcription factor called Krüppel homolog 1 (Kr-h1) (14). *Kr-h1* mRNA expression has been used as a readout of JH titers in a number of species (14–19), including *M. sexta* (20). Thus, levels of JH in an insect can be assessed by examining the expression of *Kr-h1*.

In addition to examining the *Kr-h1* expression, a useful bioassay to quantify hemolymph JH levels was developed over 40 years ago using the *black* mutant *Manduca sexta* larvae (21). The *black* mutant appears black due to excess deposition of melanin on its cuticle (22). This happens because melanin synthesis enzyme, dopa decarboxylase, is inhibited by JH (23), and the *black* mutant has a mutation that prevents production of typical amounts of JH (24). Since topical application of JH causes larvae to turn green, the color change of *black* mutant larvae can be used as a bioassay to determine the amount of JH in isolated hemolymph (21). Another potential application of the *black* mutant assay might be to determine the amount of JH-like activity (juvenoid activity) in the environment or food.

In the United States, methoprene, a JH mimic has been used commercially under the trademark name Diacon. Methoprene treatment of grains has been shown to effectively control several species of insect pests (25–28). Although studies have demonstrated the efficacy of IGR treatment on pest control, the extent to which commercially available conventional grains can impact the growth and development of laboratory insects has not been conducted.

In this study, we compared the growth of wildtype larvae, coloration of *black* mutant larvae and gene expression differences between larvae fed an artificial diet containing conventional wheat germ and one containing organic wheat germ. We also demonstrated the use of the *black* mutant larvae to determine the relative amount of juvenoid activity in conventional wheat germ. We found that conventional wheat germ extended the final larval instar. Moreover, we found that conventional wheat germ causes *black* mutant larvae to turn green with concomitant increases in the expression of the JH response gene, *Kr-h1*.

## Methods

### *M. sexta* Strains

Wildtype *M. sexta* were purchased from Carolina Biological Supply. The *black* mutant larvae arose spontaneously in a colony of wildtype *M. sexta* in the early 1970s and have been maintained by several labs. All larvae were raised on a long day photoperiod (16 h light: 8 h dark) at 26.5° C.

### Artificial Diets Used

Approximately 100 *M. sexta* were raised individually on four different diets, with ~25 larvae on each diet. The ingredients and their proportions were kept constant for each diet except for the type of wheat germ used. Two conventional brands of wheat germ were sourced from two animal diet companies, Frontier (Conventional wheat germ A) and BioServ (Conventional wheat germ B). Organic wheat germ (Lekithos), and a cornmeal (Palmetto Farms) and soy flour (NutriSoy) mix were also used as controls. The diet ingredients are listed in Supplementary Table 1.

### Growth Trajectory and *Black* Mutant Color Assay

To assess the growth trajectory of larvae, wildtype larvae were weighed daily after they reached the third instar. Measurements were terminated once they initiated the wandering stage. The wandering stage is identified by weight loss from gut purge and dorsal vessel exposure. Once gut purge initiates, larvae stop feeding and clear their guts which results in weight loss. Dorsal vessel exposure is visible as a pulsating dark line appears along the dorsal side. Larval growth rates were compared amongst the different diets. The *black* mutant larvae were reared on different diets. For controls, artificial diet containing organic wheat germ was supplemented with methoprene diluted in DMSO. All diets contained 1% DMSO. One day after the molt to the fifth instar, the color of the larvae was scored as this is the time when the larvae are darkest in color. A color scoring system (29) was used for this purpose (**Figure 3A**). JMP (SAS Institute) was used to perform one-way ANOVA and Tukey HSD *post-hoc* tests.

### Determination of *Kr-h1* Expression

The expression of *Kr-h1* in *black* mutants was assayed using quantitative real-time PCR. The epidermis of the first and second abdominal sections of larvae at the onset of a molt, just as the old cuticle of the head capsule begins to detach from the underlying new head capsule. This corresponds to the JH-sensitive period for black or green coloration (4). The epidermis was placed in Trizol (ThermoFisher), and mRNA was isolated using chloroform extraction. Subsequently, the sample was treated with DNAse (Promega) to remove any trace amount of genomic DNA. One μg of RNA was then converted to cDNA using the RevertAid First Strand cDNA Synthesis Kit (ThermoFisher). Real-time PCR was used to determine the amount of *Kr-h1* with *RpL17* serving as an internal control. For each gene, primers for each gene was mixed with SYBR Green Supermix (Bio-Rad). For *Kr-h1*, the following primers were used: 5′-GCATCGTTCACAACCTACACC-3′ (forward primer) and 5′-TCCGAGTGGAAAGCGTCAA-3′ (reverse primer) (20). For *RpL17*, the following primers were used: 5′-TCCGCATCTCACTGGGTCT-3′ (forward primer) and 5′-CACGGCAATCACATACAGGTT-3′ (reverse primer) (30). A standard curve method was used to determine the relative expression of *Kr-h1*. Four biological replicates were used with three technical replicates each. JMP (SAS Institute) was used to perform one-way ANOVA and Tukey HSD *post-hoc* tests.

## Results

### Conventional Wheat Germ Delays Metamorphosis

**Figure 1:**
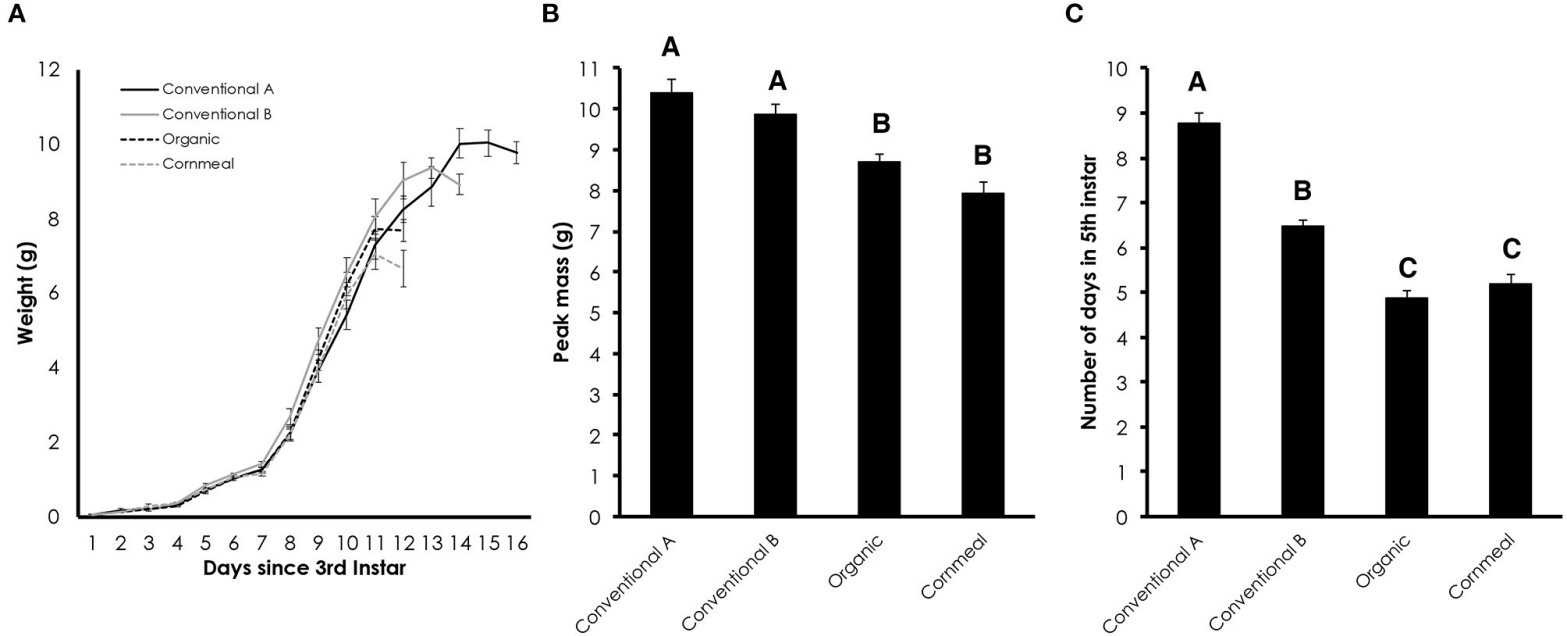
Diet containing conventional wheat germ prolongs the fifth instar duration of *M. sexta*. **(A)** Growth trajectories of larvae fed diets containing different grains. The time since the molt to the third instar is plotted. **(B)** Average peak mass of larvae fed diets containing different grains. **(C)** Number of days in the fifth instar from the first day in the fifth instar to the date of gut purge. Error bars represent standard error. Different letters represent statistically significant differences (*p* < 0.05; One-way ANOVA with post hoc Tukey HSD test).

Wildtype *Manduca* larvae were fed diets containing conventional wheat germ, organic wheat germ or cornmeal/soy flour in order to compare their growth trajectories. The cornmeal/soy flour diet serves as another control and was used to demonstrate its efficacy as a potential alternative to wheat germ. Larvae fed diets containing conventional wheat germ continued to grow past the period when the gut purge and wandering behavior normally begins in larvae fed organic and cornmeal diets (Figure 1A). This resulted in an increase in peak mass when caterpillars were reared on conventional diets [Figure 1B; One-way ANOVA: *F*_(3, 85)_ = 17.403, *p* < 0.0001]. The extended growing period caused by conventional wheat germ occurred primarily during the fifth instar [Figure 1C; One-way ANOVA: *F*_(3, 85)_ = 111.297, *p* < 0.0001], while the length of time from hatching to the fourth instar similar between diets [One-way ANOVA: *F*_(3, 85)_ = 1.993, *p* = 0.1212].

### Conventional Wheat Germ Impacts Melanic Marks of Wildtype and Inhibits Melanization in *Black* Mutant Larvae

**Figure 2:**
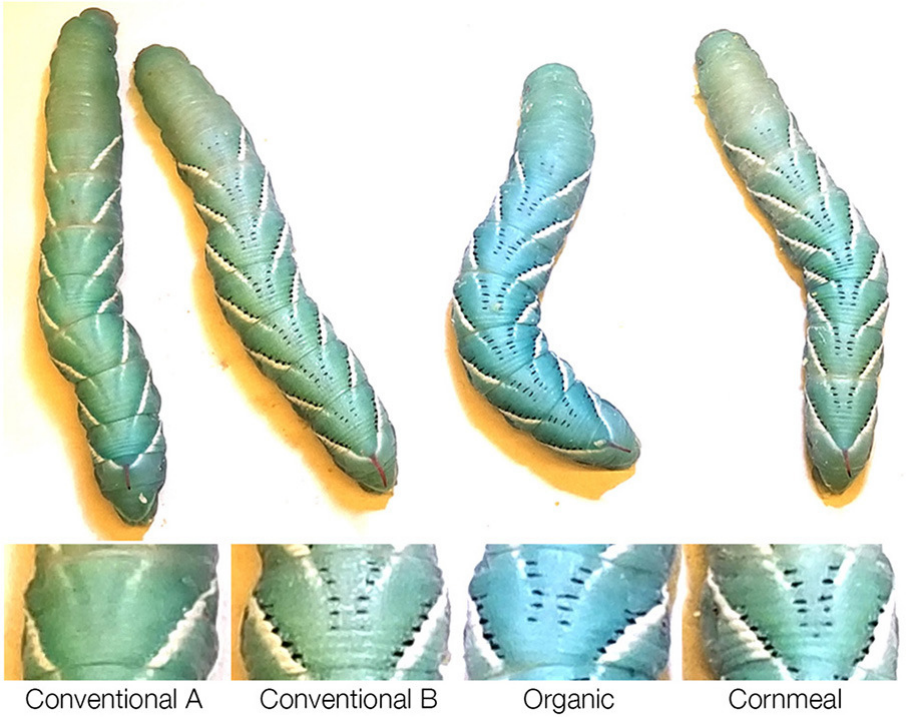
Diet containing conventional wheat germ sourced from company A eliminates black markings on the wildtype *M. sexta* larvae. (Top row) Whole body phenotype of representative fifth instar larvae reared on different diets weighing ~6 g. (Bottom row) Close-up of dorsal view of one of the segments.

**Figure 3:**
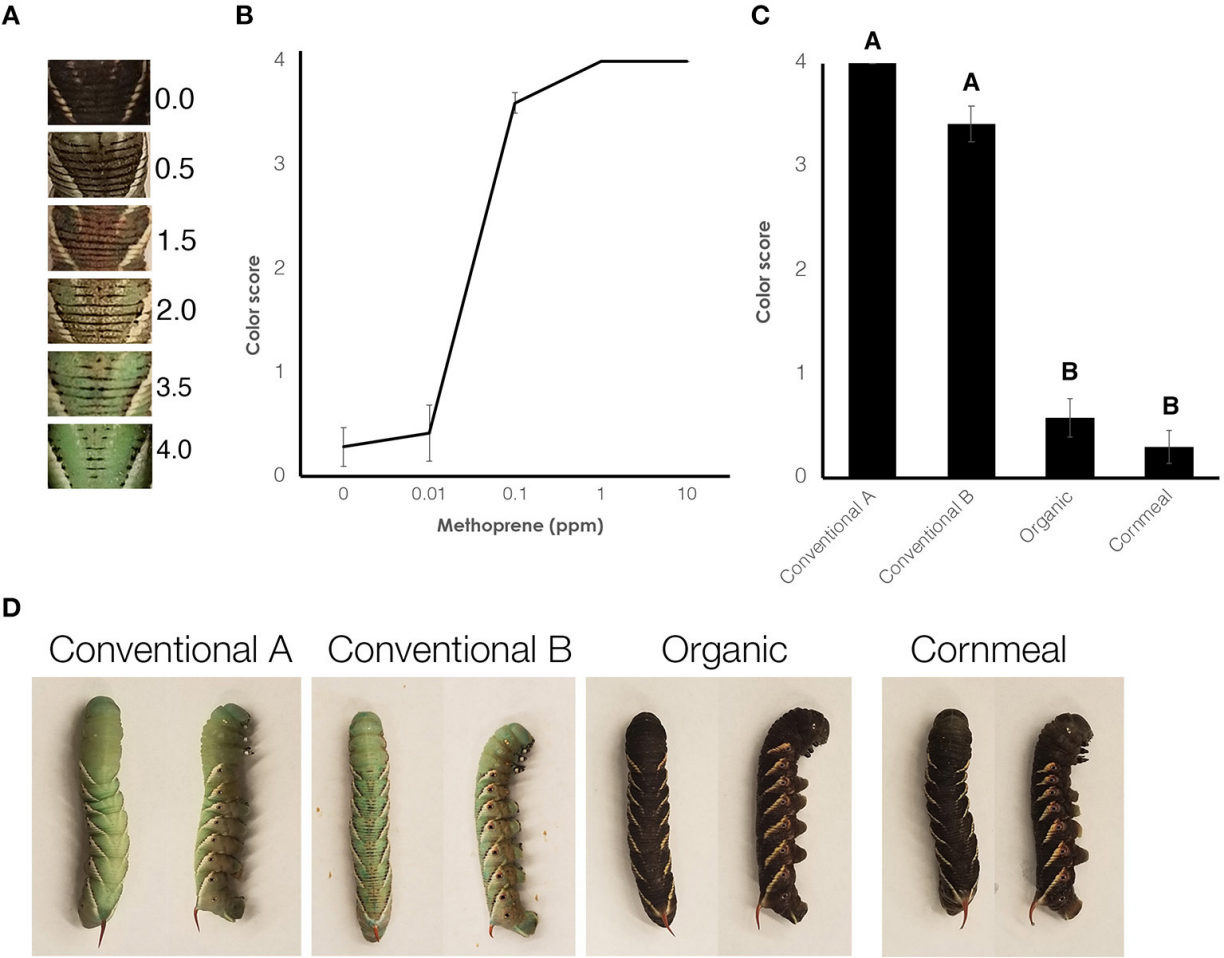
Diet containing conventional wheat germ causes *black* mutant larvae to turn green. **(A)** Scale used to score the larvae. **(B)** A dose response curve showing the average color of *black* mutants fed on diet containing organic wheat germ that has been treated with methoprene. Error bars represent standard error. **(C)** Average color of *black* mutants fed on different diets. Error bars represent standard error. Different letters represent statistically significant differences (*p* < 0.05; One-way ANOVA with *post hoc* Tukey HSD test). **(D)** Typical *black* mutant larvae fed different diets. On the left is a dorsal view; on the right is a lateral view.

While rearing the wildtype larvae, we noticed that one of the diets containing conventional wheat germ (Conventional wheat germ A) inhibited the presence of the melanic marks on the dorsal side (Figure 2). Because JH is known to inhibit melanization, we further characterized this effect by rearing *black* mutant larvae on different diets. As a control, we fed larvae on diets containing different amount of methoprene and the colors were scored (Figure 3A). Control diet containing 0.1 ppm methoprene was sufficient to cause the *black* mutant larvae to turn green (Figure 3B). In *black* mutant larvae fed the experimental diets, both diets containing the conventional wheat germ caused the larva to turn green whereas most of the larvae fed diets with organic wheat germ or cornmeal remained black [Figures 3C,D; One-way ANOVA: *F*_(3, 61)_ = 132.019, *p* < 0.0001]. These results indicate that juvenoids are present in conventional wheat germ in quantities sufficient to suppress melanin deposition.

### Conventional Wheat Germ Elevates *Kr-h1* Expression in *Black* Mutant Larvae

**Figure 4:**
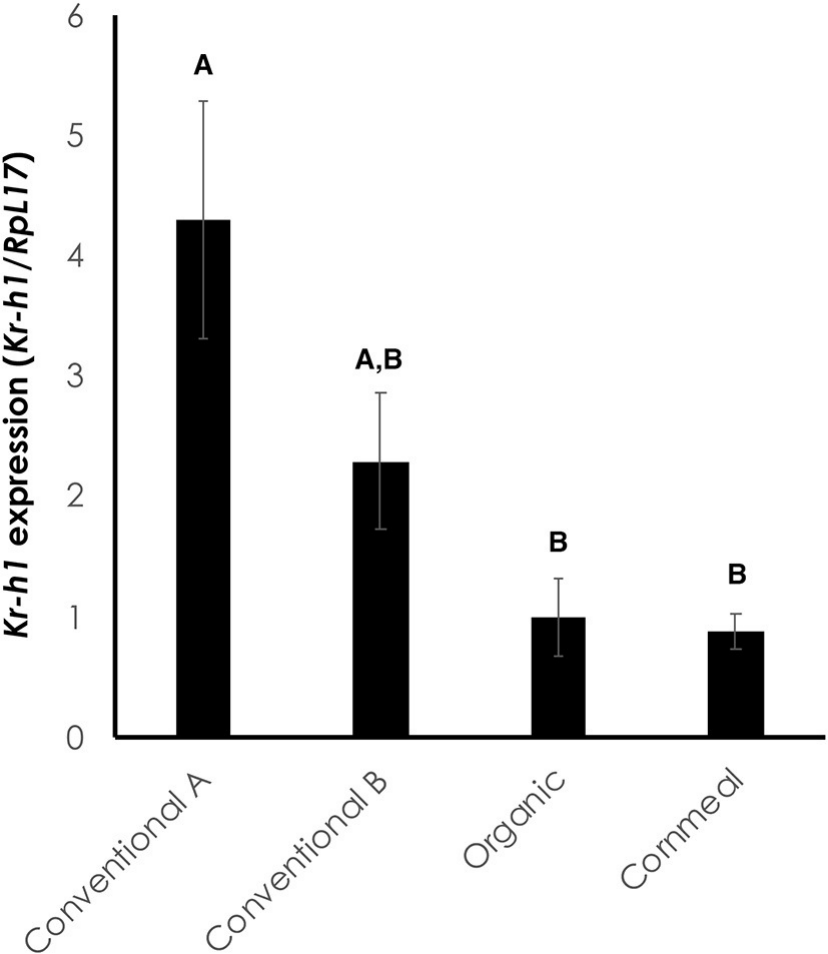
*Kr-h1* expression is elevated in the epidermis of *black* mutant larvae fed on diet containing conventional wheat germ. The epidermis was isolated at the onset of the molt to the fifth instar during the sensitive period for color determination (4). *RpL17* was used as an internal control. Each bar represents an average of four biological replicates, each with three technical replicates. Error bars represent standard error. Different letters represent statistically significant differences (*p* < 0.05; One-way ANOVA with *post hoc* Tukey HSD test).

We next sought to molecularly characterize whether the change in coloration was due to the presence of juvenoids in the diet. The expression of the JH response gene, *Kr-h1*, was significantly higher in larvae fed conventional wheat germ A compared to larvae on diets containing organic wheat germ or cornmeal/soy flour [Figure 4; One-way ANOVA: *F*_(3, 12)_ = 7.219, *p* < 0.005]. Larvae fed conventional wheat germ B had intermediate expression of *Kr-h1*. Thus, the diets which caused delays in metamorphosis and inhibited melanization also led to increased *Kr-h1* expression, indicating that the effects are likely due to the presence of juvenoids in conventional wheat germ (Figure 4).

## Discussion

In this study, we compared the impact of conventional and organic wheat germ on the growth and development of *M. sexta*. We found that conventional wheat germ caused larvae to delay metamorphosis and exhibit minor developmental changes. Using the *black* mutant larvae, we demonstrated that the conventional wheat germ contains sufficient juvenoid activity to change the color and induce the expression of the JH response gene, *Kr-h1*. The amount of juvenoid activity differed between the two conventional batches of wheat germ.

The excess duration of growth seen in the wildtype *M. sexta* that were fed conventional wheat germ stems primarily from lengthened duration of the 5th instar. This is consistent with elevated JH signaling, which prevents the production of PTTH from the brain (7). The *black* mutant assay clearly demonstrates the presence of juvenoids in the conventional wheat germ. The *black* mutant bioassay has been used to determine hemolymph titers of JH (21, 29, 31). In this study, we demonstrate the utility of this strain in determining the amount of environmental juvenoids. With the increasing use of IGRs, a sensitive indicator of environmental juvenoid activity will be increasingly important. The *black* mutant assay may be one such tool for assessing the amount of juvenoid activity in both food crops and other substrates.

IGRs have been linked to potential declines in honeybees (32). Here, we showed that laboratory insects can also be negatively impacted by IGRs used in agricultural settings. We find that physiology and gene expression can both be altered by IGRs applied to stored grains. Our work highlights the need for careful monitoring of IGRs in the environment and in dietingredients.

## Conclusions

Our study demonstrates that the use of IGRs in agricultural settings can impact the development and physiology of insects in the laboratory. We urge researchers to take caution when using stored grains from animal feed companies for mass-rearing of insects. We also urge careful monitoring of IGR levels in nature and propose that bioassays may be an inexpensive and sensitive way to monitor environmental IGR levels.

## Data Availability Statement

The original contributions presented in the study are included in the article/Supplementary Material. Further inquiries can be directed to the corresponding author.

## Author Contributions

JF, JT, and YS contributed to conception and design of the study, carried out the experiments, and wrote sections of the manuscript. All authors contributed to manuscript revision, read, and approved the submitted version.

## Funding

This work was funded by the National Science Foundation grant IOS-2002354 to YS and funds provided by Wellesley College.

## Conflict of Interest

The authors declare that the research was conducted in the absence of any commercial or financial relationships that could be construed as a potential conflict of interest.

## Publisher's Note

All claims expressed in this article are solely those of the authors and do not necessarily represent those of their affiliated organizations, or those of the publisher, the editors and the reviewers. Any product that may be evaluated in this article, or claim that may be made by its manufacturer, is not guaranteed or endorsed by the publisher.
