# Supplementary material for: Conventional and Organic Wheat Germ Have Distinct Physiological Effects in the Tobacco Hornworm, Manduca Sexta: Use of Black Mutant Assay to Detect Environmental Juvenoid Activity of Insect Growth Regulators
Source: Front Insect Sci. 2021 Oct 21;1:744847. doi: 10.3389/finsc.2021.744847 (PMC10926489; doi:10.3389/finsc.2021.744847)
Supplement: Supplementary file 1 [file Data_Sheet_1.PDF]

Supplemental Table 1. Diet ingredients used to make 1L of *Manduca* diet. Conventional and organic diets based off of Yamamoto 1969; cornmeal/soy flour amounts based off of Bennett and White 1989.

INGREDIENTS	Conventional Diet A	Conventional Diet B	Organic Diet	Cornmeal diet
Distilled water	1 L	1 L	1 L	1 L
Conventional wheat germ (Frontier)	107.6 g			
Conventional wheat germ (BioServ)		107.6 g		
Organic wheat germ (Lekithos)			107.6 g	
Cornmeal (Palmetto Farms)				78 g
Soy Flour (NutriSoy)				42 g
Gelcarin	15.7 g			
Raw linseed oil	5.38 ml			
36.5% formalin	3.14 ml			
Casein	48.4 g			
Sucrose	43.0 g			
Torula yeast	21.5 g			
Cholesterol	4.71 g			
Wessons salt	16.1 g			
Sorbic acid	2.7 g			
Methyl paraben	1.3 g			
Ascorbic acid	6.7 g			
Streptomycin	0.27 g			
Kanamycin	0.07 g			
Nicotinic acid	13.5 mg			
Riboflavin	6.73 mg			
Thiamine	3.14 mg			
Pyriodoxine	3.14 mg			
Folic acid	3.14 mg			
Biotin	0.27 mg			

References

Bennett, R.R. &White, R.H. (1989) Influence of carotenoid deficiency on visual sensitivity, visual pigment and P-face particles of photoreceptor membrane in the moth Manduca sexta. J Comp Physiol A *164*, 321–31.

Yamamoto , R.T. (1969) Mass rearing of the tobacco hornworm. II. Larval rearing and pupation. J Econ Entomol *62*, 1427-1431.
